# The influence of Gamification on medical students’ diagnostic decision making and awareness of medical cost: a mixed-method study

**DOI:** 10.1186/s12909-023-04808-x

**Published:** 2023-10-28

**Authors:** Kosuke Ishizuka, Kiyoshi Shikino, Hajme Kasai, Yoji Hoshina, Saito Miura, Tomoko Tsukamoto, Kazuyo Yamauchi, Shoichi Ito, Masatomi Ikusaka

**Affiliations:** 1 Department of General Medicine, Chiba University Hospital, 1-8-1, Inohana, Chuo-ku, Chiba-city, Chiba pref Japan; 2 Department of General Medicine, Yokohama City University School of Medicine, Yokohama, Kanagawa Japan; 3 Department of Community-oriented Medical Education, Graduate School of Medicine, Chiba University, Chiba, Japan; 4 Health Professional Development Center, Chiba University Hospital, Chiba, Japan; 5 Department of Respirology, Graduate School of Medicine, Chiba University, Chiba, Japan; 6 Department of Medical Education, Graduate School of Medicine, Chiba University, Chiba, Japan; 7 Department of Psychiatry, Chiba University Hospital, Chiba, Japan

**Keywords:** Clinical reasoning, Decision making, Diagnostic error, Gamification, Medical cost

## Abstract

**Background:**

The gamification of learning increases student enjoyment, and motivation and engagement in learning tasks. This study investigated the effects of gamification using decision-making cards (DMCs) on diagnostic decision-making and cost using case scenarios.

**Method:**

Thirty medical students in clinical clerkship participated and were randomly assigned to 14 small groups of 2–3 medical students each. Decision-making was gamified using DMCs with a clinical information heading and medical cost on the front, and clinical information details on the back. First, each team was provided with brief clinical information on case scenarios. Subsequently, DMCs depending on the case were distributed to each team, and team members chose cards one at a time until they reached a diagnosis of the case. The total medical cost was then scored based on the number and contents of cards drawn. Four case scenarios were conducted. The quantitative outcomes including confidence in effective clinical decision-making, motivation to learn diagnostic decision-making, and awareness of medical costs were measured before and after our gamification by self-evaluation using a 7-point Likert scale. The qualitative component consisted of a content analysis on the benefits of learning clinical reasoning using DMCs.

**Result:**

Confidence in effective clinical decision-making, motivation to learn diagnostic decision-making, and awareness of medical cost were significantly higher after the gamification. Furthermore, comparing the clinical case scenario tackled last with the one tackled first, the average medical cost of all cards drawn by students decreased significantly from 11,921 to 8,895 Japanese yen. In the content analysis, seven advantage categories of DMCs corresponding to clinical reasoning components were extracted (information gathering, hypothesis generation, problem representation, differential diagnosis, leading or working diagnosis, diagnostic justification, and management and treatment).

**Conclusion:**

Teaching medical students clinical reasoning using DMCs can improve clinical decision-making confidence and learning motivation, and reduces medical cost in clinical case scenarios. In addition, it can help students to acquire practical knowledge, deepens their understanding of clinical reasoning, and identifies several important clinical reasoning skills including diagnostic decision-making and awareness of medical costs. Gamification using DMCs can be an effective teaching method for improving medical students’ diagnostic decision-making and reducing costs.

**Supplementary Information:**

The online version contains supplementary material available at 10.1186/s12909-023-04808-x.

## Background

Clinical reasoning is a core competency for all health-care professionals; therefore, it is critical for medical students to develop clinical reasoning skills [1, 2]. The process of clinical reasoning is a series of steps that include selecting and visiting a medical institution when a patient has a health problem, gathering information when the patient consults a medical professional, and making a tentative diagnosis [3]. In this process, history-taking, physical examination, clinical tests, referrals, and consultations are conducted, and a clinical decision/diagnosis is made. In addition, clinical reasoning has a context-specific nature [4]. For example, a physician can make two different diagnostic decisions despite examining two patients with the same chief complaint and similar history and physical examination findings [4].

Competencies that are important for teaching clinical reasoning can be categorized into five domains, each of which requires specific knowledge, skills, and behaviors [5]. These domains are: (1) clinical reasoning concepts, (2) history and physical examination, (3) choosing and interpreting diagnostic tests, (4) problem identification and management, and (5) shared decision-making. It is important to promote the acquisition of effective clinical reasoning skills for each of these processes by designing a curriculum with a specific purpose in terms of what, how, and when they are taught [3, 5]. However, clinical reasoning is challenging for many novice students owing to inadequate knowledge, poor data collection skills, and inappropriate approaches to information processing [1].

Although it is important for medical students to learn the process of clinical reasoning, it is also important for them to learn the components such as communication skills, relationship of mutual trust among health-care professionals, evidence-based practice, reasoning outside of the medical context, patient-physician relationship and rapport with the patient, clinical skills of data collection (history-taking, physical examination, specific procedural skills), critical thinking, consideration of medical costs, explicit reliance on baseline probabilities, appropriate use of algorithms, visual-based diagnosis, and cognitive styles [6]. Furthermore, although diagnosis is a major component of the clinical reasoning process, it is important for students to develop management and decision-making skills that take into account various additional factors such as resources and cost-effectiveness [5–7]. Curbing medical expenses is a pressing issue in any country, but it is especially important to raise awareness in Japan, where the universal health insurance system and universal access provide patients with easy access to medical care, which has led to a high frequency of medical consultations [8, 9]. In addition, in order to shorten the time required for a single consultation, diagnosis by laboratory or radiological examination, rather than using time-consuming medical interviews and physical examination, has become the norm [9–11]. This is a major reason for the increase in medical costs, and research has shown that both patients and doctors in Japan have a low level of awareness of medical costs [9–11]. A previous study of residents and clinical fellows in Japan reported that displaying fees at the time of ordering clinical tests in paper-based simulated cases resulted in cost reduction [9]. Intervention studies and education, audit and feedback, system-, incentive- or penalty-based interventions have been shown to be effective in increasing awareness of medical costs in several countries [12–16].

Although it is significant to acquire clinical reasoning skills through self-study, emphasis should also be placed on developing these skills using in-depth case studies [17]. The increasing use of technology to supplement learning resources for students in problem-based learning has recently attracted much attention in many areas of gamification [18]. Gamification is defined as “the use of game design elements in non-game contexts.” [19]. The gamification of learning increases student enjoyment, and motivation and engagement in learning tasks [18, 20, 21]. In addition, the usefulness of gamification in clinical reasoning education for health care professional education has been reported to date [22, 23].

The purpose of this study is to investigate the effect of gamification using decision-making cards (DMCs) on diagnostic decision-making and awareness of medical costs in the clinical reasoning education of medical students.

## Methods

### Mixed-methods research

To integrate quantitative and qualitative evaluation, a mixed-methods study was conducted using an exploratory sequential design [24–26]. This type of research study design takes advantage of the strengths of each type of study design and minimizes their shortcomings. Furthermore, it allows the researchers to understand the experimental results better by incorporating medical students’ perspectives. This is based on the US National Institutes of Health guidelines, which advocate a mixed-methods approach to research “to improve the quality and scientific power of data” and better address the complexity of issues in health science education [27, 28].

### Study design

A cross-sectional study was conducted using case scenarios to investigate the effects of gamification using DMCs on diagnostic decision-making and awareness of medical costs among medical students as a component of their clinical reasoning education. The quantitative outcomes included students’ confidence in effective clinical decision-making, motivation to learn diagnostic decision-making, and awareness of medical costs. In addition, the correctness of the final diagnosis was scored, and the total number of cards drawn, and the total medical cost were recorded.

A qualitative evaluation was conducted to examine the cognitive aspects of the medical students, which is thought to influence the learning effectiveness of clinical reasoning by gamification using DMCs.

The results of the quantitative and qualitative components were integrated as a mixed-methods, sequential explanatory study [24–26]. The qualitative data were collected using an open-ended questionnaire, and content analysis was used to investigate the advantages of clinical reasoning education for medical students through gamification using DMCs.

### Participants

This study was conducted at a single facility in the Department of General Medicine of Chiba University Hospital in Japan. The study included all 30 medical students (two fourth-year medical students, 26 fifth-year medical students, and two sixth-year medical students) at the Chiba University School of Medicine who participated in a clinical clerkship (CC) in our department in November and December 2019. This study was embedded in their CC rotation in the department; thus, the participants were not sampled randomly. Additionally, it was conducted with medical students in the year of study in which they participated in CC. Therefore, we included students from different study years. Ahmad et al. reported that individual and small-group settings are ideal for gamification because they enhance students’ interest, effort, and motivation [29]. In addition, peer-assisted learning, which is defined as learning through matched-status individuals from “similar social groupings who are not professional teachers,” has been shown to improve medical students’ learning of clinical information and skills [30]. Therefore, in this study, considering these backgrounds and the number of participants, they were randomly assigned to 14 small-group teams, with each team consisting of 2–3 medical students. The gamification using DMCs for four case scenarios was implemented and the order in which the case scenarios were assigned to each team was randomized. All participants had already received lectures and simulation training in basic and clinical medicine by the fourth year and a pre-CC objective structured clinical examination as one of examinations for promotion to CC together with the Computer-Based Test, which is another assessment of medical knowledge applied before CC. Students who were unable to participate in one of the case scenarios for any reason were excluded from the study. We had to suspend this study because of the interruption in CCs by the COVID-19 pandemic. Therefore, we only analyzed the gathered data up till then.

### Procedure

**Fig. 1:**
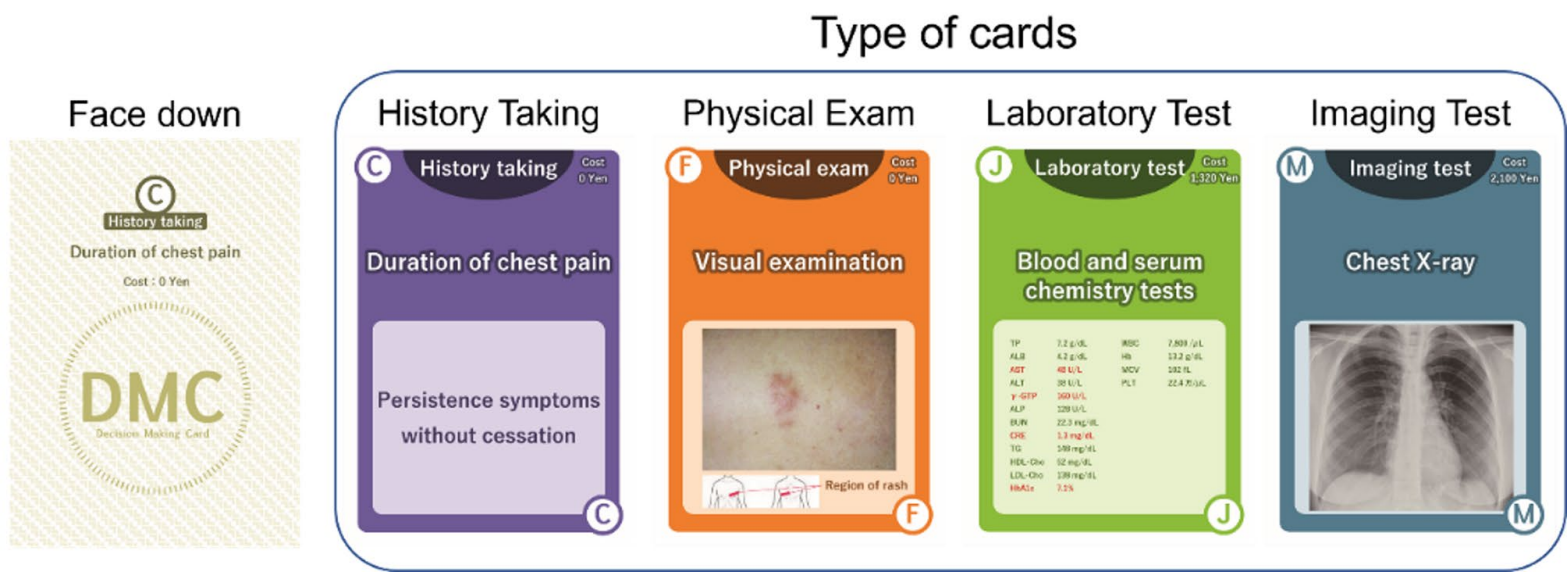
Type of cards on DMC

**Fig. 2:**
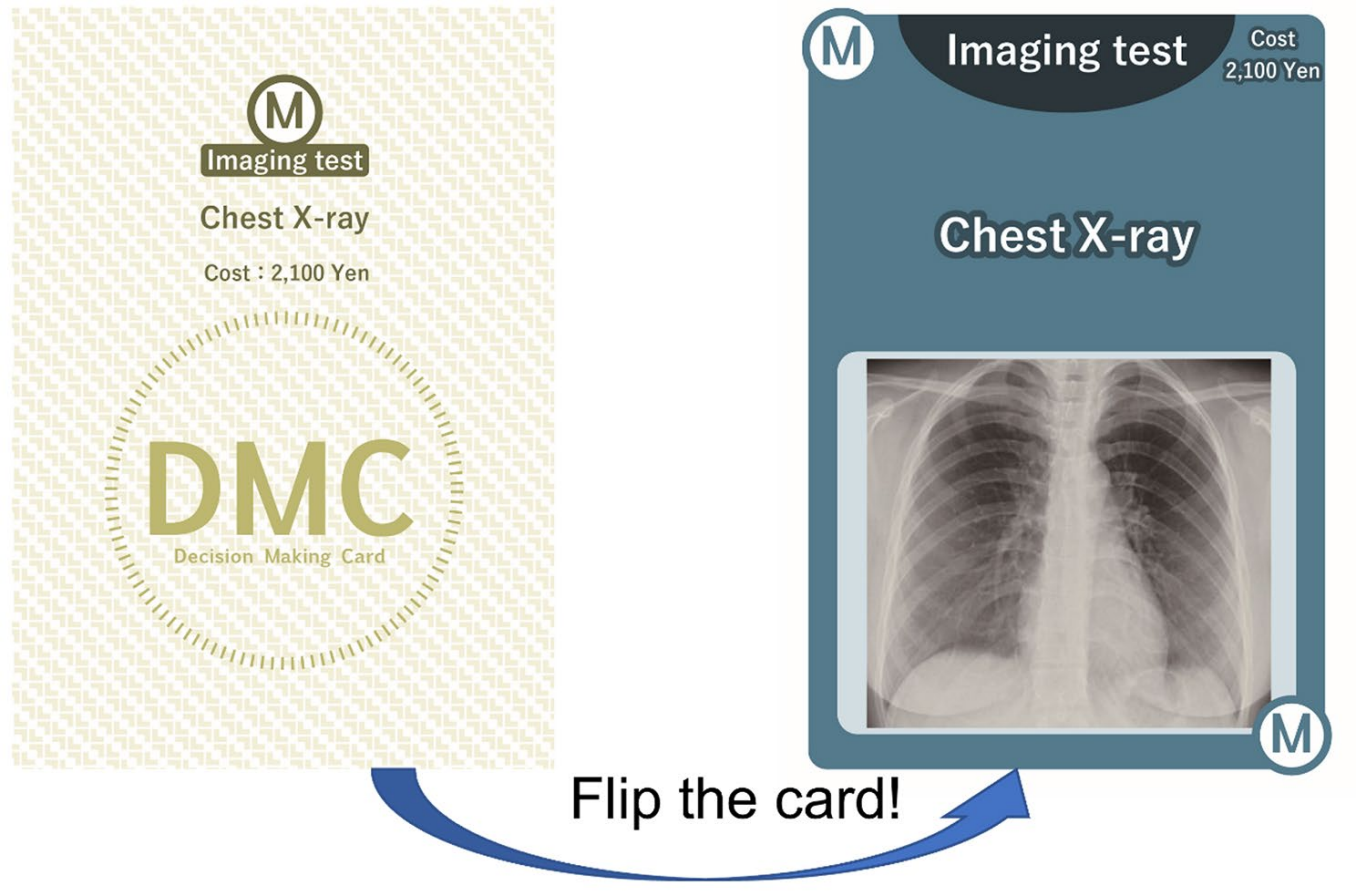
The front and back of DMC cards The DMCs had a clinical information heading and medical cost on the front. In addition, these cards had the details of clinical information on the back. A letter of the alphabet on the DMC card was used as the card identifier.

The use of the DMCs was gamified. The DMCs had a clinical information heading and medical cost on the front of the card, and clinical information details on the back of the card (Figs. 1 and 2). First, each team was provided with brief clinical information on each case scenario (Supplement 1). DMCs were then distributed to each team according to the case scenario, and team members chose cards one at a time until they reached a diagnosis (Supplement 2). There was no limit to the number of times that a card could be drawn. The medical costs were calculated in Japanese yen [JPY]. (According to the foreign exchange rates on January 27, 2023, 1 JPY = 0.0062 Great Britain pound [GBP], 0.0077 US dollars [USD], or 0.0071 euro [EUR].) The four case scenarios were chest pain (herpes zoster), dyspnea (panic disorder), back pain (ureteral calculus), and abdominal pain (diverticulitis). The total number of cards and total medical costs for each case scenario are shown in Table 1.

**Table 1:** The total number of cards and total medical costs per each case scenario

	Medical history (Number of cards)	Physical examination (Number of cards)	Clinical tests (Number of Cards)	Total number of cards (Number of cards)	Total medical costs (JPY)
Chest pain (Herpes zoster)	5	4	5	14	22,400
Dyspnea (Panic disorder)	4	6	6	16	29,620
Back pain (Ureteral calculus)	5	7	6	18	43,000
Abdominal pain (diverticulitis)	6	3	5	14	24,820

The medical costs were calculated in Japanese yen [JPY]. (According to the foreign exchange rates on January 27, 2023, 1 JPY = 0.0062 Great Britain pound [GBP], 0.0077 US dollars [USD], or 0.0071 euro [EUR].)

Five faculty members (KI, KS, HK, YH, and SM) were randomly assigned to supervise the four case scenarios, with at least one faculty member assigned to each case scenario. Before conducting the gamification, each faculty member was given instruction on the case scenarios and the contents of the gamification. All instructions were standardized and were given immediately before the gamification (Supplement 3). Gamification using DMCs followed the simulation education methods with the steps of briefing, simulation, and debriefing. The faculty members briefed the students in advance to clarify the purpose of gamification using DMCs. The faculty members provided the following briefing (explanation of the rules before the start). “Teams of two or three students will be challenged with the problem.” “You will have 10 minutes to respond. When the time is up, the timekeeper will give you instructions.” “Question and answer sheets will be distributed. Answer sheets will be collected after the completion of the session. You may write notes on the answer sheet. Do not write on the question paper, as it will be used by other groups.” “Points are awarded for each correct diagnosis, and points are deducted for each additional card drawn.” “Please fill in the name of your group on the answer sheet and wait until the signal to begin.” During the gamification, which lasted approximately 10 min. students learned by using the DMCs independently under the supervision of the faculty members, without any intervention or lecture by the faculty members. Immediately after the gamification, the faculty members debriefed the students on the process of selecting the cards for approximately 10 min. The correct answers were given during the debriefing, and the students and the instructor reflected on the reasoning process. In addition, the diagnosis, number of cards drawn, the order in which the cards were drawn, and the appropriateness of the total medical cost were reviewed. Furthermore, the faculty members were able to give an example of the process model. Each faculty member was adequately skilled to explain the process of clinical reasoning to the participating students.

**Fig. 3:**
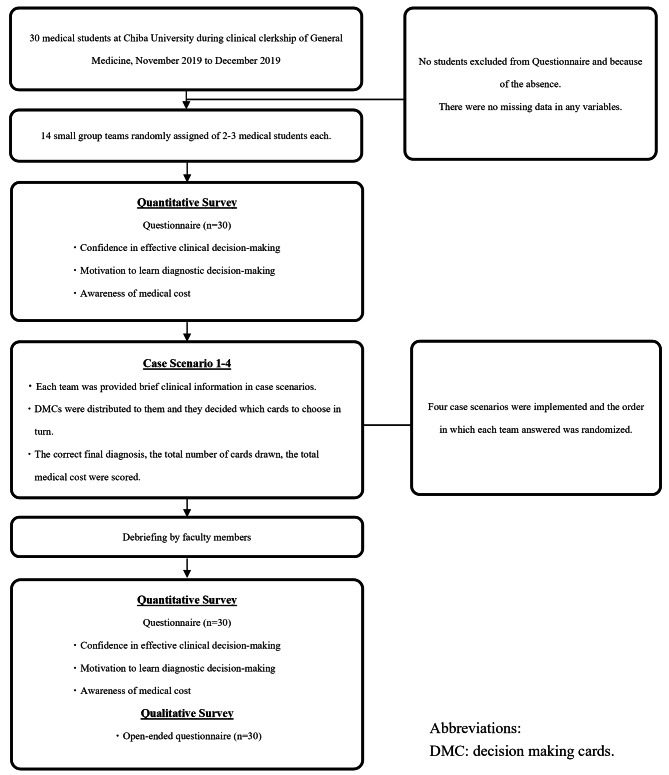
Flow diagram of the design

For each group, the gamification using DMCs was implemented using the four case scenarios. The order in which the case scenarios were assigned to each group was randomly assigned equally (Fig. 3). The case scenarios were selected through focus group discussion by two supervisors of the Department of General Medicine and one supervisor of Respiratory Medicine at the University (KI, KS, and HK). The four case scenarios of chest pain (herpes zoster), dyspnea (panic disorder), back pain (ureteral calculus), and abdominal pain (diverticulitis) were conducted (Table 1). The four case scenarios were selected because they present challenges in pattern recognition and can be used to assess analytical and diagnostic-reasoning skills. In Japan, the fourth version of the national core curriculum for undergraduate medical education, released in 2016, introduced a new list of possible diagnoses for 37 common signs, symptoms, and pathophysiological findings that ought to be learned as part of the six-year undergraduate curriculum [31]. Regarding these common signs, students must acquire the competence to anticipate a set of differential diagnoses from the earliest phase of the diagnostic process, and should gather information to confirm or refute an initial hypothesis, select and perform the relevant history-taking and physical examination, and interpret the findings to confirm or refute the initial hypothesis [31, 32]. The four case scenarios were selected to include any one of the 37 common signs in the Japanese National Medical Examination questions.

### Intervention data

#### Making the correct final diagnosis, number of cards drawn, and medical cost

Each group calculated the total medical cost for each case scenario, and we compared the percentage making the correct final diagnosis, the total number of cards drawn, and the total medical costs for the first and last clinical case scenario exercises.

### Outcome measures

#### Confidence in effective clinical decision-making, motivation to learn diagnostic decision-making, and awareness of medical cost

The confidence in effective clinical decision-making, motivation to learn diagnostic decision-making, and awareness of medical cost were evaluated before and after the gamification using a 7-point Likert scale ranging from 1 (“strongly disagree”) to 7 (“strongly agree”). The content of the questionnaire survey was decided through focus group discussion by two supervisors of the Department of General Medicine and one supervisor of Respiratory Medicine at the University (KI, KS, and HK).

### Sample size

As this study also served as an educational program for a CC in our department, medical students who were assigned to the rotation at the beginning of the study period were included in the study. For the quantitative data, the sample size required for the Wilcoxon signed-rank test of the difference between the means of the two groups was calculated to be 28 students in total, assuming a significance level of 0.05, a power of 0.8, and an effect size of 0.5. However, we had to suspend this study because of the COVID-19 pandemic and only analyzed the data collected prior to the study suspension. Therefore, a total of 30 students distributed across 14 groups were included in the analysis.

### Data analysis

All statistical analyses were performed using SPSS Statistics for Windows 26.0 (IBM Co., Armonk, NY, USA) with a significance level of less than 5%. The quantitative data are expressed as mean ± standard deviation (SD) unless otherwise indicated. The correct final diagnosis, the total number of cards drawn, and the total medical cost were compared using the Wilcoxon signed-rank test. We also compared confidence in effective clinical decision-making, motivation to learn diagnostic decision-making, and awareness of medical cost before and after the gamification using the Wilcoxon signed-rank test.

### Qualitative survey

Following the quantitative evaluation, the qualitative evaluation was conducted to evaluate the cognitive aspects of the intervention on medical students. Gamification using DMCs is thought to enhance the learning effectiveness of clinical reasoning [24–26, 33]. The results of the quantitative and qualitative evaluations were integrated as a mixed-methods sequential explanatory study [24–26, 33]. An open-ended questionnaire, designed according to the study objectives, was used to investigate the advantages of clinical reasoning education through gamification using DMCs [28, 33]. The content of the questionnaire survey was decided through discussions among the faculty members (KI and KS) [28]. Medical students were asked the following open-ended questions: “What are the advantages of gamification using DMCs? Why do you think so?” [28, 33]. All 30 medical students who participated in the gamification using DMCs answered the questionnaire [28]. Names and other identifiers were removed from the questionnaire and the statements were tabulated [28]. The faculty members did not reveal their personal attitudes and behaviors to the students [28, 33]. A team debrief was held after the questionnaire survey [28, 33]. There were no repeat questionnaire surveys, and participants were not asked to review the transcripts or to provide feedback [28, 33].

Content analysis was used to analyze the response categories in the qualitative research (Table 2) [28, 33–35]. A preliminary analytic template was developed as a starting point for analysis [28, 33–35] Two researchers (KI, KS) independently read all open-ended questionnaire transcripts and performed the initial coding [28, 33–35]. To ensure the quality of the research, researcher triangulation was conducted by two researchers (KI and KS), who discussed, identified, and agreed on the coding of the descriptors [28, 33–35]. Following the coding, similar codes were grouped into categories and subcategories, which were regularly discussed and reviewed by a third researcher, HK (who had experience in qualitative research), to ensure the credibility of the findings [28, 33–35]. The findings were reported using the consolidated criteria for reporting qualitative research (COREQ) checklist [35].

**Table 2:** The step of qualitative content analysis

Gamification	
Step	Description
Step 1: Overview	A preliminary analytic template was developed as a starting point for analysis. All 30 medical students were included in the qualitative analysis. A coding system was developed. - 1. Open coding: A smaller sample of the documents was read. Viewpoints and contexts were written and labeled. General categories were generated that bundled multiple labels. - 2. Axial coding: Entire sample of documents was reviewed. Specific passages belonging under categories identified in initial open coding were tagged. - 3. Selective coding: The researcher combed through the documents in search of miscoded passages and discrepant evidence.
Step 2: Independent analysis	Two researchers (KI, KS) independently read and performed the initial coding of all open-ended questionnaire transcripts. They compiled descriptors and categories for analysis. The coding system was then used to generate categories for the study. After coding, similar codes were grouped into categories and subcategories. The categories were linked to the major findings of the study.
Step 3: Discussion of categories	To ensure the quality of the research, researcher triangulation was conducted by two researchers (KI, KS), who discussed, identified, and agreed on the coding of the descriptors.
Step 4: Interpretation and verification	Following the coding, similar codes were grouped into categories and subcategories, which were regularly discussed and reviewed by HK (who had experience in qualitative research) to ensure the credibility of the findings and interpret of meaning derived from the study. The consolidated criteria for reporting qualitative research (COREQ) checklist was used to report the findings.
Step 5: Comparison and theory	The findings were compared with relevant literature and theory. Implications for future research and reform were outlined.

The analytic categories were set according to the seven working definitions for the different components of clinical reasoning (information gathering, hypothesis generation, problem representation, differential diagnosis, leading or working diagnosis, diagnostic justification, and management and treatment) (Supplement 4) [36]. After open coding, similar codes were classified into subcategories and categories. We analyzed the concepts in each of the seven clinical reasoning components and calculated the number of analysis units for each concept [36]. We also grouped similar codes as categories and checked the clinical reasoning components to which they corresponded.

### Ethics statement

This research was performed in accordance with the Declaration of Helsinki and approved by the Ethics Review Committee of the Chiba University Graduate School of Medicine (Chiba, Japan) on May 7, 2019 (approval number: 3425). The study procedures were explained to the medical students, and informed consent for participation was obtained. Although the researcher who administered the consent was also a class teacher, it was made clear to the medical students that participation in this study would not affect their grade evaluations. This study was registered in the University Hospital Medical Information Network Clinical Trials Registry (UMIN-CRT) (UMIN000049765).

## Results

### Participant characteristics

All 30 eligible students (2 fourth year medical students (6.7%), 26 fifth year medical students (86.7%), and 2 sixth year medical students (6.7%)) provided informed consent and were included in both the quantitative and qualitative components of the evaluation. The mean age of the students was 23.9 years (standard deviation: 2.3 years) and 19 of the 30 students (63.3%) were male. There were no missing data.

### Making the correct final diagnosis, number of cards drawn, and medical cost

The percentage of students making the correct final diagnosis, total number of cards drawn, total medical cost of the case scenarios did not differ significantly between the first and last clinical case scenario exercises (71% and 43%, p = 0.157, r = 0.378; 5.6 ± 2.1 and 6.2 ± 4.7, p = 0.825, r = 0.059; and 30,351 ± 8,710 JPY and 29,569 ± 7,774 JPY, p = 0.825, r = 0.059, respectively). However, the average medical cost of all cards drawn by students decreased significantly between the first and last clinical case scenario exercises (from 11,921 ± 8,895 JPY to 8,699 ± 13,167 JPY, p = 0.046, r = 0.411).

### Confidence in effective clinical decision-making, motivation to learn diagnostic decision-making, and awareness of medical cost

**Fig. 4:**
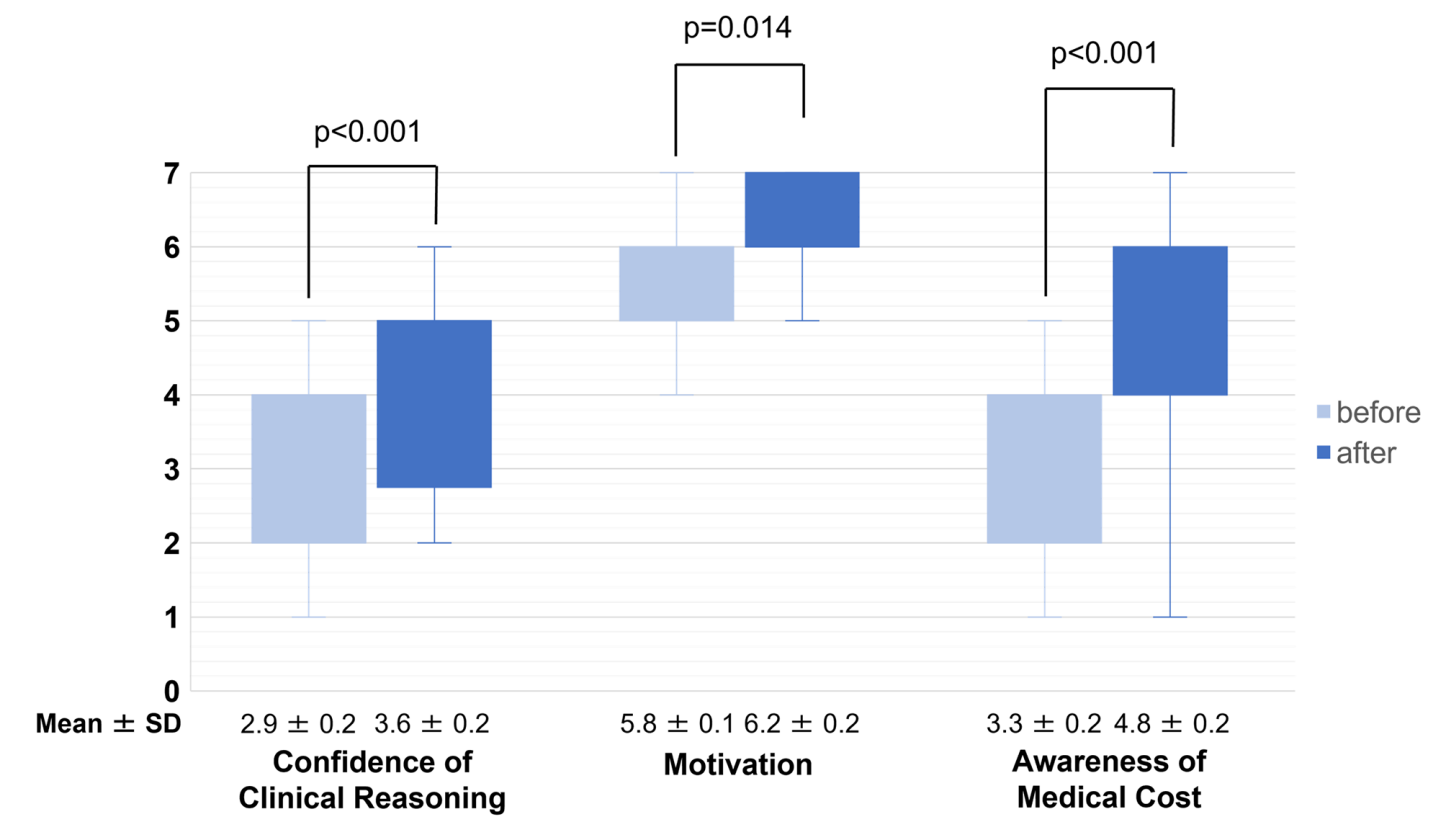
Confidence in effective clinical decision-making, motivation to learn diagnostic decision-making, and awareness of medical cost The scale of 7-point Likert scale was 1: strongly disagree, 7: strongly agree. SD: standard deviation.

Confidence in effective clinical decision-making, motivation to learn diagnostic decision-making, and awareness of medical cost were significantly increased after participating in the gamification than before (2.9 ± 0.2 to 3.6 ± 0.2, p < 0.001, r = 0.697 5.8 ± 0.1 to 6.2 ± 0.2, p = 0.014, r = 0.448, 3.3 ± 0.2 to 4.8 ± 0.2, p < 0.001, r = 0.685, respectively) (Fig. 4).

### Content analysis

Informed consent was obtained from all 30 medical students who were subjected to the quantitative evaluation. All 30 participants were included in the qualitative analysis. The categories of analysis were set according to the seven working definitions for the different components of clinical reasoning (Supplemental 4) [36]. After analyzing the records of all 30 medical students, we confirmed that we had reached thematic saturation.

Table 3 shows the categories, subcategories, number of codes, and representative quotations. A total of 92 codes were generated from the open-ended questionnaire. We identified seven categories and 24 subcategories of advantages of clinical reasoning education by gamification using DMCs, covering all seven clinical reasoning components [36]. Furthermore, in the subcategories of content analysis, “listing differential diagnosis” was the most frequent subcategory, followed by “awareness of medical costs,” “clinical features of diseases,” “narrowing down the differential diagnosis,” and “generating differential diagnoses.”

**Table 3:** Absolute frequencies of codes for each category

Category	Subcategory	Quotes
**Information gathering (15)**	Clinical features of disease (9)	*“Gamification allows medical students to learn clinical features of common diseases.”*
	Methods of clinical information gathering (2)	*“After going through gamification, I think medical students can appreciate the importance of history taking.”*
	Physical examination (2)	*“Gamification makes medical students realize the importance of information gathering on the physical examination.”*
	Appropriate medical history-taking to gather information (1)	*“One good thing about gamification is that it allows medical students to practice taking an appropriate medical history to gather information.”*
	Case specificity in clinical reasoning (1)	*“An advantage of gamification is that medical students can learn that there are points to consider in each case.”*
**Hypothesis generation (11)**	Generating differential diagnoses (6)	*“I am confident that I can generate clinical hypotheses from the patient’s chief complaint, thanks to the gamification.”*
	Hypothesis-driven information gathering (5)	*“Gamification helps medical students to learn the importance of performing the appropriate clinical tests according to the clinical hypothesis.”*
**Problem representation (8)**	Cognitive bias (5)	*“I was able to learn actively. Thanks to gamification, I think that we can realize the terror of falling into cognitive bias.”*
	Priority of clinical information (2)	*“An advantage of gamification is that it allows medical students to learn how to prioritize clinical information. This made for a more serious learning experience than usual about clinical reasoning.”*
	Priority of physical examination (1)	*“Gamification helps medical students learn the concept of prioritizing the history taking and physical examination.”*
**Differential diagnosis (16)**	Listing differential diagnoses (16)	*“Gamification was a lot of fun. An advantage of gamification is that it increases one’s ability to list differential diagnoses from symptoms.”*
**Leading or working diagnosis (17)**	Awareness of medical costs (11)	*“It was fun, like a game. Gamification helps medical students to realize the importance of being aware of the medical costs.”*
	Priority of diagnostic testing (3)	*“Gamification allows medical students to realize the importance of considering the relative importance of history taking, physical examination, and clinical tests according to the differential diagnosis.”*
	Cost-effectiveness of diagnostic testing (1)	*“Gamification made medical students realize the importance of training. because it is difficult to reach a diagnosis by the shortest path and at the lowest medical costs.”*
	Under-adaptation to clinical examination (1)	*“Gamification makes medical students more aware that fixed medical costs makes it harder to perform clinical tests.”*
	Examination procedures (1)	*“Thanks to gamification, I enjoyed learning about clinical reasoning. Gamification motivates medical students to learn about real-life examination procedures.”*
**Diagnostic justification (21)**	Narrowing down the differential diagnosis (8)	*“It was fun to learn in a game-like atmosphere. Gamification helps medical students learn how to narrow down the differential diagnosis.”*
	Diagnostic error (5)	*“Gamification was an interesting learning experience. Gamification can teach medical students about diagnostic errors.”*
	Definitive diagnosis process (3)	*“An advantage of gamification is that medical students can learn in depth about the process of making a definitive diagnosis.”*
	Sensitivity and specificity of clinical examination (3)	*“Gamification allows medical students to learn about the concept of sensitivity and specificity of each test.”*
	False-positive test results (2)	*“Medical students can learn through gamification that clinical tests can have false positives.”*
**Management and treatment (4)**	Appropriate management and treatment (2)	*“Gamification motivates medical students to learn more about treatment and management according to differential diagnosis.”*
	Exclusion of critical disease (1)	*“Gamification gives medical students the skills to rule out acute diseases.”*
	Decision-making in real time (1)	*“One advantage of gamification is that it teaches the importance of clear decision-making.”*

The values shown in curved brackets after each category and subcategory are the number of codes in that category/subcategory

The subcategories “clinical features of diseases” (9 codes), “methods of clinical information gathering” (2 codes), “physical examination” (2 codes), “appropriate medical history-taking to gather information” (1 code), and “case specificity in clinical reasoning” (1 code) were grouped in the category “information gathering” (Total 15 codes).

‘*Gamification allows medical students to learn clinical features of common diseases*.’ (ID = 11).

The subcategories “generating differential diagnoses” (6 codes), and “hypothesis-driven information gathering” (5 codes) were grouped in the category “hypothesis generation” (Total 11 codes).

‘*I am confident that I can generate clinical hypotheses from the patient’s chief complaint, thanks to the gamification*.’ (ID = 2).

The subcategories “cognitive bias” (5 codes), “priority of clinical information” (2 codes), and “priority of physical examination” (1 code) were grouped in the category “problem representation” (Total 8 codes).

‘*I was able to learn actively. Thanks to gamification, I think that we can realize the terror of falling into cognitive bias*.’ (ID = 22).

The subcategory “listing the differential diagnosis” (16 codes), was assigned to the category “differential diagnosis” (Total 16 codes).

‘*Gamification was a lot of fun. The advantage of gamification is that it increases students’ ability to list differential diagnoses from symptoms*.’ (ID = 9).

The subcategories “awareness of medical costs” (11 codes), “priority of diagnostic testing” (3 codes), “cost-effectiveness of diagnostic testing” (1 code), “under-adaptation to clinical examination” (1 code) and “examination procedures” (1 code) were grouped into the category “leading or working diagnosis” (Total 17 codes).

‘*It was fun, like a game. I think that the gamification helps medical students realize the importance of being aware of the medical costs*.’ (ID = 8).

The subcategories “narrowing down the differential diagnosis” (8.codes), “diagnostic error” (5 codes), “definitive diagnosis process” (3 codes), “sensitivity and specificity of clinical examinations” (3 codes), and “false-positive test results” (2 codes) were grouped into the category “diagnostic justification” (Total 21 codes).

‘*It was fun to learn with a game-like atmosphere. I think that gamification helps medical students learn how to narrow down the differential diagnosis*.’ (ID = 21).

The subcategories “appropriate management and treatment” (2 codes), “exclusion of critical disease” (1 code), and “decision-making in real time” (1 code) were classified into the category “management and treatment” (Total 4 codes).

‘*Gamification motivates medical students to learn more about treatment and management according to the differential diagnosis*.’ (ID = 13).

## Discussion

This study suggests that gamification using DMC may be considered an effective educational method for improving medical students’ diagnostic decision-making ability and their awareness of medical costs in the clinical reasoning process. Comparing the first and last clinical case scenario exercises, the average medical cost of all cards drawn by students decreased significantly. In addition, confidence in effective clinical decision-making, motivation to learn diagnostic decision-making, and awareness of medical cost were significantly higher after than before the gamification.

Gamification has been reported to improve motivation and engagement with learning tasks, produce positive learning outcomes through increased enjoyment, and improve clinical care [18, 20, 21]. The quotes from the qualitative survey in this study also showed that the medical students perceived factors such as “enjoy learning,” “sense of fun and games,” “active learning,” and “serious learning” as advantages of gamification using DMCs in addition to the seven clinical reasoning components. Another advantage of gamification may be that students can simulate the decision-making process by imagining real patients and real situations in clinical settings, although the common benefit of using a case-based approach is considered [37]. There are various subtypes of gamification, which are based on a combination of attributes such as skill, strategy, and chance [37]. Learning with card and board games, defined by the layout of the game, improves medical students’ communication skills and promotes active interaction learning with other players [38, 39]. Therefore, gamification using DMCs is likely to stimulate the decision-making process, which is one of the most important processes of clinical reasoning, and bring positive learning effects to medical students. In line with self-determination theory, game design elements can be used to enhance learners’ feelings of relatedness, autonomy, and competence to foster their intrinsic motivation [40]. However, these basic psychological needs may be undermined by the over-justification effect and the negative effects of competition if they are not consistent with the objectives of gamification [40]. Adding game design elements to increase extrinsic motivation can adversely impact learners who already have a strong intrinsic motivation because of over-justification owing to overreliance on external motivating factors can result in a net negative effect on engagement and motivation [40, 41]. Consideration of the potential for either negative or positive effects on motivation is key in choosing which systems to gamify, which game design elements to use, and which students are most likely to benefit [40]. In addition, the negative effects of competition may result in a deficit of trust among fellow learners, and a loss of motivation to learn among low-ranking learners, and when there is no change in ranking [40, 42]. Steps to minimize the negative effects of competition include maximizing collaborative opportunities (e.g., team-based competition) [40]. Also, the percentage of students making the correct final diagnosis did not differ significantly between the first and last clinical case scenario exercises. The possible reason was considered that the evaluation of clinical reasoning has highly case-specificity elements [43].

It is important to clearly understand the advantages and disadvantages of gamification, to take a cautious approach when integrating gamification, to discuss comprehensive learning objectives between teachers and students, and for the teacher to provide feedback to the students [40]. In this study, the briefing and debriefing by the faculty members were used to clarify the significance and learning objectives of gamification using DMCs [40]. In addition, gamification using DMCs is an easy-to-implement educational method because it can be easily created from existing cases and the cards can be printed on both sides. Therefore, gamification using DMCs may provide an educational opportunity to teach medical students clinical reasoning skills.

The quantitative and qualitative data of this study showed that teaching medical students clinical reasoning using DMCs as a gamification method, led to improved clinical decision-making confidence and learning motivation in clinical case scenarios. In addition, it helped students to acquire practical knowledge, deepened their understanding of clinical reasoning, and identified several important clinical reasoning skills, including diagnostic decision-making.

Conversely, although diagnosis is an important part of the clinical reasoning process, it is also important for students to develop management and decision-making skills in this process, taking into account various factors such as resources and cost-effectiveness [5, 7]. In Japan, clinical reasoning education with an awareness of medical costs is important because of increasing medical costs and a low level of awareness of medical costs among physicians and patients [9]. In this study, awareness of medical costs increased significantly after the gamification, and the average medical cost of all cards drawn by the students decreased significantly from the first to the last clinical case scenario exercise. Furthermore, among the subcategories of the content analysis, “listing differential diagnoses” and “awareness of medical costs” were the first and second more frequently mentioned, suggesting that the intervention was effective at teaching clinical reasoning with an awareness of medical costs. Therefore, gamification using DMCs of case scenarios appears to be an effective educational method for reducing medical costs and teaching clinical reasoning with awareness of medical costs.

### Limitations

This study has several limitations. First, it was conducted using scenario tasks with paper-based materials, not actual patients. Although this study revealed the usefulness of gamification using DMCs for teaching clinical reasoning to medical students, it is necessary to verify whether the advantages of gamification using DMCs for teaching clinical reasoning are similar for real patients. Second, the qualitative study of the present study revealed that teaching clinical reasoning to medical students through gamification using DMCs is effective in identifying some important skills related to clinical reasoning. However, it could not separate the effect of gamification using DMCs from the effect of using a case-based approach and increased interaction with faculty members on improving students’ competence in clinical reasoning such as reflection. Third, the lack of a control group is a limitation. Cook and Beckman reported that showing a significant difference of an educational intervention without a control group only demonstrates that learning can occur [44]. Furthermore, this study revealed that the average total cost was significantly reduced in the last case scenario exercise compared to the first, but further comparative verification of the average cost using a control group that is not shown the cost is needed. Fourth, in this study, the clinical tests were limited to those related to the diagnosis determined in the focus group discussion, and not necessarily those obtained by broader consensus. The required clinical tests may differ depending on whether the evaluation includes treatment, and on the practices in each country. In actual clinical practice, it is necessary to consider the characteristics and evidence of each clinical test, treatment guidelines, and discussions among medical professionals including specialists in charge of diagnosis and treatment to decide which tests are necessary. Fifth, this study was conducted on medical students at a single institution and department in Japan. Therefore, the results of this study may not be generalizable beyond the specific population from which the sample was drawn. Further validation is needed to determine whether the results can be applied to residents and general physicians. Sixth, in this study, participants were randomly assigned to 14 small-group teams, with each team consisting of 2–3 medical students. However, the learning effects of gamification may vary according to differences in group size. Seventh, quantitative outcomes gauged before and after educational intervention were anchored in self-assessment. Eighth, the questionnaire’s content was formulated through focus group discussions involving two supervisors from the Department of General Medicine and one from Respiratory Medicine at the university (KI, KS, and HK). Ninth, the reliability and validity of the survey haven’t been ascertained.

## Conclusions

Teaching medical students clinical reasoning using DMCs can improve clinical decision-making confidence and learning motivation and reduces medical cost in clinical case scenarios. In addition, it can help students acquire practical knowledge, deepens their understanding of clinical reasoning, and be trained in several important clinical reasoning skills, including diagnostic decision-making and awareness of medical costs. Gamification using DMCs can be effective at reducing medical costs in clinical case scenarios and educating medical students in clinical reasoning with awareness of medical costs.

### Electronic supplementary material

Below is the link to the electronic supplementary material.

Supplementary Material 1

Supplementary Material 2

Supplementary Material 3

Supplementary Material 4

## Data Availability

The raw dataset supporting the conclusions of this article is available from the corresponding author upon request.
