# Supplementary material for: The influence of Gamification on medical students’ diagnostic decision making and awareness of medical cost: a mixed-method study
Source: BMC Med Educ. 2023 Oct 28;23:813. doi: 10.1186/s12909-023-04808-x (PMC10613361; doi:10.1186/s12909-023-04808-x)
Supplement: Supplementary file 1 — Supplementary Material 1 [file 12909_2023_4808_MOESM1_ESM.docx]

**Supplement 1. Example case scenario: chest pain (herpes zoster)**

**Information Sheet**

*Please read the following information sheet and fill in your responses on the response form.*

| **Setting:** Outpatient clinic (family practice)  **Date:** Saturday, March 16, 2019, 9 AM  **Patient:** 68-year-old man  **Chief complaint:** Chest pain  **Present medical history:** The patient came to the clinic with a chief complaint of left chest pain that started around 7 AM that morning.  **Medical History:** Hypertension, dyslipidemia  **Medications:** Amlodipine (Ca channel blocker) 10 mg/day, Pitavastatin (HMG-CoA reductase inhibitor) 2 mg/day  **Family history:** Father had a myocardial infarction  **Taste:** 20 cigarettes/day × 48 years; alcohol consumption: 2 g of sake daily  **Vital signs:** Blood pressure 148/78mmHg, body temperature 36.8°C, pulse 90/min (normal), respiratory rate 12 breaths/min, SpO_2_ 99%.  **Level of consciousness:** Clear |
| --- |

**Question 1**

Please state the number of cards that you selected.

**Question 2**

Please list the order of the cards that you selected.

**Question 3**

Please specify the most likely diagnosis.
