# Supplementary figures and images for: The influence of Gamification on medical students’ diagnostic decision making and awareness of medical cost: a mixed-method study
Source: BMC Med Educ. 2023 Oct 28;23:813. doi: 10.1186/s12909-023-04808-x (PMC10613361; doi:10.1186/s12909-023-04808-x)

**Supplement 2. The scene of gamification using DMCs**

**
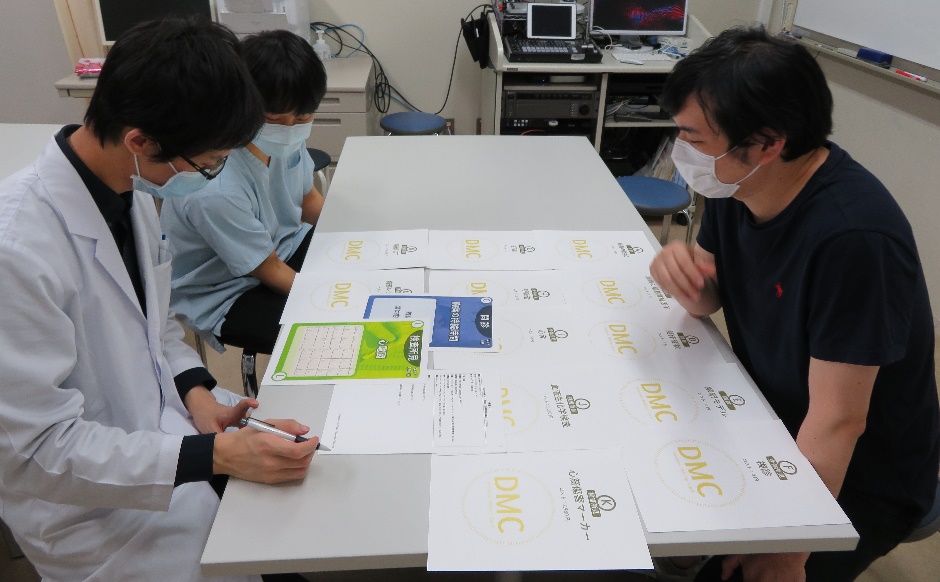
**

Student 2

Student 1

Supervisor

Supplement: Supplementary file 2 — Supplementary Material 2 [file 12909_2023_4808_MOESM2_ESM.docx]
