# Supplementary material for: The influence of Gamification on medical students’ diagnostic decision making and awareness of medical cost: a mixed-method study
Source: BMC Med Educ. 2023 Oct 28;23:813. doi: 10.1186/s12909-023-04808-x (PMC10613361; doi:10.1186/s12909-023-04808-x)
Supplement: Supplementary file 3 — Supplementary Material 3 [file 12909_2023_4808_MOESM3_ESM.docx]

**Supplement 3. The flow of gamification**

**Before the session starts**

- 1. Set DMCs face down.
- 2. Set the question paper face down (1 sheet).
- 3. Set the question number paper face down (1 sheet).
- 4. As soon as the students arrive at the designated area, provide the following briefing (explanation of the rules before the start).
- “Teams of two or three students will be challenged with the problem.”
- “You will have 10 minutes to respond. When the time is up, the timekeeper will give you instructions.”
- “Question and answer sheets will be distributed. Answer sheets will be collected after the completion of the session. You may write notes on the answer sheet. Do not write on the question paper, as it will be used by other groups.”
- “Points are awarded for each correct diagnosis, and points are deducted for each additional card drawn.”
- “Please fill in the name of your group on the answer sheet and wait until the signal to begin.”

**During the session**

- Debrief the student group on the case.
- During the session, the evaluations of the participating groups are written on the evaluator's sheet for the instructor.
- When the response forms are collected, they are checked for completeness.

**After the session**

- Check and collect the response forms from the students to make sure that they are complete.
- Compile the sheets as a set with the evaluator's sheet.
- Debriefing (10 minutes).
- The correct answers are given, and the students and the instructor reflect on the thinking process.
- The diagnosis, number of cards drawn, the order in which the cards were drawn, and the appropriateness of the total medical cost are reviewed.
- It is also a good idea to give an example of the instructor's process model.
- After the debriefing, wait for the signal from the timekeeper and instruct the participants to move to the next session (changeover time: 2 minutes).
- During the changeover, prepare for the next scenario (back before the session starts).

Four sessions were conducted, as described above. After the four sessions had been completed, the answer sheets and evaluator sheets were collected.

Abbreviations: DMC: decision making cards.
