# Supplementary material for: The influence of Gamification on medical students’ diagnostic decision making and awareness of medical cost: a mixed-method study
Source: BMC Med Educ. 2023 Oct 28;23:813. doi: 10.1186/s12909-023-04808-x (PMC10613361; doi:10.1186/s12909-023-04808-x)
Supplement: Supplementary file 4 — Supplementary Material 4 [file 12909_2023_4808_MOESM4_ESM.docx]

**Supplement 4. Working definitions for the different components of clinical reasoning**^36^

| **Component** | **Definition** |
| --- | --- |
| **Information gathering** | The process of acquiring the data needed to generate or refine hypotheses. This is usually an active process that includes taking a history, performing a physical, acquiring lab or radiographic data, reviewing the medical record, etc., but may be implicit (through observation) as well. The selection of information to gather is driven by knowledge representations of disease (i.e., scripts, schema). |
| **Hypothesis generation** | An early nonanalytic or analytic process by which a physician tries to find diseases that can explain a patient’s clinical findings. Hypothesis generation involves activation of knowledge representations of disease in an iterative process that feeds back on information gathering and vice versa (e.g., hypothesis generation leads to more information gathering, which leads to more hypothesis generation and/or refinement). |
| **Problem representation** | A dynamic mental representation of all the relevant aspects of the case (including the patient’s clinical findings, biopsychosocial dimensions, etc.) that can be communicated in a summary that includes semantic qualifiers and key findings. |
| **Differential diagnosis** | A list of diagnostic hypotheses that represent the best summary categorizations of the problem representation (Note: Different specialties may have different priorities when it comes to ordering the differential; e.g., in emergency medicine, life-threatening diseases are often listed first, whereas in internal medicine, the most likely diseases are usually listed first). As the strength of confidence and evidence for these representations change, a leading diagnosis emerges. |
| **Leading or working diagnosis** | A diagnosis for which a physician’s probability of a given disease has crossed his or her threshold to pursue additional testing or to initiate treatment, even if the diagnosis is not definitive. |
| **Diagnostic justification** | The attempt to use the evidence (key clinical findings) from information gathering to choose one or more diagnoses as most likely and to defend that choice, comparing and contrasting other possible diagnoses. Justification often involves communication (orally or in writing) when socially required and may not be part of the a priori clinical reasoning process. |
| **Management and treatment** | The actions that follow the clinical reasoning process, including prognostication, management, treatment, prevention strategies, and palliation of symptoms (including improvement of quality of life) and justification for such actions. |
